# Skin wetness perception across body sites in children and adolescents aged 7–16 years old

**DOI:** 10.1113/EP092691

**Published:** 2025-05-31

**Authors:** Alessandro Valenza, Hannah Blount, Jade Ward, Charlotte Merrick, Riley Wootten, Jasmin Dearden, Charlotte Wildgoose, Antonino Bianco, Alex Buoite‐Stella, Victoria L. Filingeri, Peter R. Worsley, Davide Filingeri

**Affiliations:** ^1^ ThermosenseLab, Skin Sensing Research Group, School of Health Sciences The University of Southampton Southampton UK; ^2^ Sport and Exercise Sciences Research Unit, SPPEFF Department University of Palermo Palermo Italy; ^3^ Clinical Unit of Neurology, Department of Medicine, Surgery and Health Sciences Trieste University Hospital‐ASUGI University of Trieste Trieste Italy; ^4^ Psychological and Behavioural Sciences, School of Psychology University of Derby Derby UK; ^5^ Pressurelab, Skin Sensing Research Group, School of Health Sciences The University of Southampton Southampton UK

**Keywords:** adolescents, body temperature regulation, children, elderly, skin, thermal sensation, wetness perception

## Abstract

Human skin wetness perception relies on the multisensory integration of thermal and mechanical cues during contact with moisture. Yet, it is unknown whether children and adolescents perceive skin wetness similarly to younger and older adults. We investigated skin wetness perceptions across the forehead, neck, forearm, and foot dorsum in 12 children/adolescents (4F/8M; 12 ± 3 years), 41 younger (21F/20M; 25 ± 3 years), and 21 older adults (11F/10M; 56 ± 6 years), during two established quantitative sensory tests. Our results indicated that, given the same moisture content (0.8 mL of water), very cold‐wet stimuli applied to the forearm were perceived by all groups as wetter than neutral‐wet (mean difference: 35.5 mm on a 100‐mm visual analogue scale for wetness [95% CI: 22.3, 38.7]; *P *< 0.0001; ∼35% difference) and very hot‐wet stimuli (mean difference: 22.7 mm [95% CI: 14.5, 40.9]; *P *< 0.0001; ∼23% difference). Children/adolescents also reported greater wetness perceptions than older adults during cold‐wet stimulation of the forehead, neck and foot dorsum (mean difference: 20.6 mm; 95% CI: 1.5, 39.7; *P* = 0.031; ∼21% difference). In all age groups, the foot dorsum presented higher cold‐wet sensitivity (mean difference: 11.1mm [95%CI 2.2, 20.0] p = 0.010; ~11% difference) and lower warm‐wet sensitivity than the neck (mean difference: 12.9mm [95%CI 2.8, 23.0] p = 0.008; ~13% difference). We conclude that wetness perceptions in children/adolescents (age range: 7–16 years) are similar to those of adults in that both present (1) a characteristic U‐shaped relationship between stimulus temperature and perceived wetness magnitude and (2) similar body regional patterns. These findings provide novel evidence on age‐dependent variations in wetness perception which could inform user‐centred innovation in thermal protection and garment design.

## Abstract (highlights)

- **What is the central question of this study?**
  Do children and adolescents perceive skin wetness similarly to younger and older adults?
- **What is the main finding and its importance?**
  We found that wetness perception in children and adolescents (age range: 7–16 years) was similar to wetness perception in adults in that both presented (1) a characteristic U‐shaped relationship between stimulus temperature and perceived wetness magnitude as well as (2) similar body regional patterns. These findings provide novel evidence on age‐dependent variations in wetness perception which could inform user‐centred innovation in thermal protection and garment design.

## 1 INTRODUCTION

Whether arising from sweat running over our skin or from grasping a cold wet glass of water, the perception of skin wetness, i.e. *hygrosensing*, is one of the most common sensory experiences in our lives (Filingeri & Havenith, 2018). Humans are very sensitive to skin wetness, and people can discriminate wetness levels differing by as little as of 0.04 mL of water (Ackerley et al., 2012); yet there is no evidence that our skin possesses a hygroreceptor (Filingeri & Havenith, 2015). Over the past ∼10 years, our group have shown that both younger and older, otherwise healthy adults, as well as individuals with clinical conditions (e.g. people affected by multiple sclerosis) perceive physical wetness on their skin by integrating cooling‐related, thermosensory cues (induced by conductive and evaporative heat transfer in the presence of moisture on the skin) in combination with tactile and mechanosensory cues (arising from the movement of moisture across the skin) (Christogianni et al., 2022; Filingeri et al., 2014a, 2014b, 2015a; Valenza et al., 2019; Wildgoose et al., 2021). As a result, we have strong evidence leading us to believe that the perception of wetness is a phenomenon of the central nervous system, resulting from higher‐order neural structures optimally integrating multisensory thermal (e.g. cold) and tactile (e.g. stickiness) inputs arising from the skin's contact with moisture (Filingeri et al., 2014a).

The observation that younger and older adults exhibit optimal integration of multisensory mechanisms for hygrosensing opens a series of fundamental biological questions on how these mechanisms evolve in the developing brain. Yet, there is no empirical evidence that indicates how and when in our development we learn to integrate the multisensory cues (i.e. thermal, tactile and visual (Merrick et al., 2023a, 2023b) that we use to infer the presence of wetness on the skin. Touch is the first sense to develop in humans, and it provides the sensory foundation upon which we develop awareness of our body and surroundings (Bremner & Spence, 2017). Touch also plays a pivotal role in multisensory perceptual development (Bremner & Spence, 2017). There is evidence that optimal multisensory integration in children does not reach the adult level until after 8 years of age (Ernst, 2008). Before this age, the ongoing development of individual sensory modalities (e.g. touch and vision) seems to preclude Bayesian‐like multisensory integration (Gori et al., 2008). Nevertheless, children as young as 3 years old can describe an object they touch as wet (Flavell et al., 1989). Notwithstanding that humans may perceive skin wetness during early development, it remains unknown whether children and adolescents perceive skin wetness across their body similarly to younger and older adults (Christogianni et al., 2022; Filingeri et al., 2014a; Valenza et al., 2019; Wildgoose et al., 2021).

Our findings have expanded the understanding of the neurophysiology of human skin wetness perception in younger and older adults (Filingeri & Havenith, 2018). But how hygrosensing develops during childhood (and declines in later life) and how changes in the sensitivity to skin wetness across the lifespan impact on humans’ ability to effectively interact with our surrounding environments remain unknown. This knowledge gap opens a series of fundamental biological questions on somatosensory development and ageing, which have clear implications to support innovation in healthcare and user‐centred wearables (Valenza et al., 2019), and to inform public health policies to protect vulnerable groups (e.g. children, elderly) from increasingly frequent heatwaves (Ebi et al., 2021).

The aim of this study was to investigate whether children and adolescents perceive skin wetness similarly to younger and older adults. Due to the paucity of data on this topic, we decided to test healthy children and adolescents across a wide age range (i.e. 7–16 years old), and to compare their perceptual responses with those of a large cohort of younger and older, otherwise healthy, male and female participants. We hypothesized that these children and adolescents may present a relationship between stimulus temperature and perceived wetness magnitude like that observed in adults; furthermore, we hypothesized that children may also present regional differences in wetness perception across the body, aligned to those observed in adults.

## 2 METHODS

### 2.1 Ethical approval

The testing procedure and the conditions were explained to each participant, and they all gave written informed consent for participation. The data presented in this study are the result of several data collection campaigns performed by our Thermosense Laboratory between August 2018 and February 2023, during which we employed the same methodology across different participant cohorts. The studies were approved by the Loughborough University Ethics Sub‐Committee for Human Participants (no. R18‐P083), the Research Integrity and Governance team of University of Southampton (ERGOII 72799; ERGOII 78327), and the University of Trieste Ethics Committee (Ref. 068_2020H #COVID19#). Testing procedures were in accordance with the tenets of the *Declaration of Helsinki* (note, the studies were not registered in a database).

### 2.2 Participants

Considering the paucity of data on children's wetness perception which could have informed a sample size calculation, we opted to recruit a convenience sample of 12 children/adolescents (4F/8M, age range: 7–16 years; mean age: 12.1 ± 2.6 years; body mass: 43.2 ± 12.9 kg; height: 1.58 ± 0.16 m) with whom to evaluate wetness perception responses. Data from children/adolescents were then compared with data from two participant groups, i.e. a younger adult cohort of 41 individuals (21F/20M, age range: 20–34 years; mean age: 25 ± 3.9 years; body mass: 71.3 ± 12.7 kg; height: 1.73 ± 0.1 m) and an older cohort of 21 individuals (11F/10M, age range: 45–65 years; mean age: 55.2 ± 6.4 years; body mass: 71.5 ± 15 kg; height: 1.72 ± 0.09 m). Data from the younger and older adult cohorts were collected over several years as part of larger experiments in adults, using the same methods used in the children/adolescent tests.

All participants had no history of neurological and skin‐related conditions (e.g. eczema), and they were recruited from the student and general populations of the University of Southampton (UK), Loughborough University (UK) and University of Trieste (Italy).

Female participants self‐reported the day of their menstrual cycle at the time of testing. Female participants of the children group (*n* = 4) reported no commencement of menarche. Female participants of the young adult group (*n* = 21) reported to be spread across a typical 28‐day menstrual cycle (day of cycle: 18 ± 10), with two of them reporting irregular periods and only five of them taking oral contraceptives at the time of the study. Regarding female participants of the older adult group (*n* = 11), four self‐reported having regular periods (day of cycle: 20 ± 6); the remaining seven participants self‐reported to be menopausal (i.e. no longer having regular periods for at least 6 months). Amongst the seven menopausal participants, four of them reported being under hormone replacement therapy and one of them to be taking hormonal contraception.

Participants were instructed to refrain from: (1) performing strenuous exercise in the 48 h preceding testing and (2) consuming caffeine or alcohol in the 24 h preceding testing.

### 2.3 Experimental design

All participants took part in one testing session, during which we performed two separate experiments: (1) a multisensory integration test and (2) a body mapping test of skin wetness perception. These were based on our established quantitative sensor testing protocols (Filingeri et al., 2014a; Valenza et al., 2019). The first experiment was conceived to evaluate the relationship between stimulus temperature and perceived wetness magnitude across the lifespan (which is indicative of multisensory integration mechanisms); the second experiment aimed to evaluate differences in wetness perception across the body. All experiments were conducted at rest and in a thermoneutral environment (ambient temperature: 23.2 ± 1.6°C; relative humidity: 39.5 ± 4.8%). As with previous studies (Filingeri et al., 2014a, 2014c, 2018), all participants were blinded to the nature and application of the stimuli in each test to limit expectation biases.

#### 2.3.1 Multisensory integration test

Six stimuli varying in temperature (i.e. ranging from 10°C below to 10°C above local skin temperature) and moisture content (i.e. either dry or saturated with 0.8 mL of water) were applied to the volar surface of the forearm in a randomized order (i.e. single 10‐s application at midpoint between wrist and antecubital fossa, with 30‐s interval between stimuli) using a hand‐held temperature‐controllable probe (surface area: 1.32 cm^2^). Specifically, the six stimuli corresponded to:

1. Very hot wet stimulus (10°C above local skin temperature and saturated with 0.8 mL of water).
2. Warm wet stimulus (5°C above local skin temperature and saturated with 0.8 mL of water).
3. Neutral wet stimulus (equal temperature as local skin temperature and saturated with 0.8 mL of water).
4. Neutral dry stimulus (equal temperature as local skin temperature and dry).
5. Cold wet stimulus (5°C below local skin temperature and saturated with 0.8 mL of water).
6. Very cold wet stimulus (10°C below local skin temperature and saturated with 0.8 mL of water).

**FIGURE 1:**
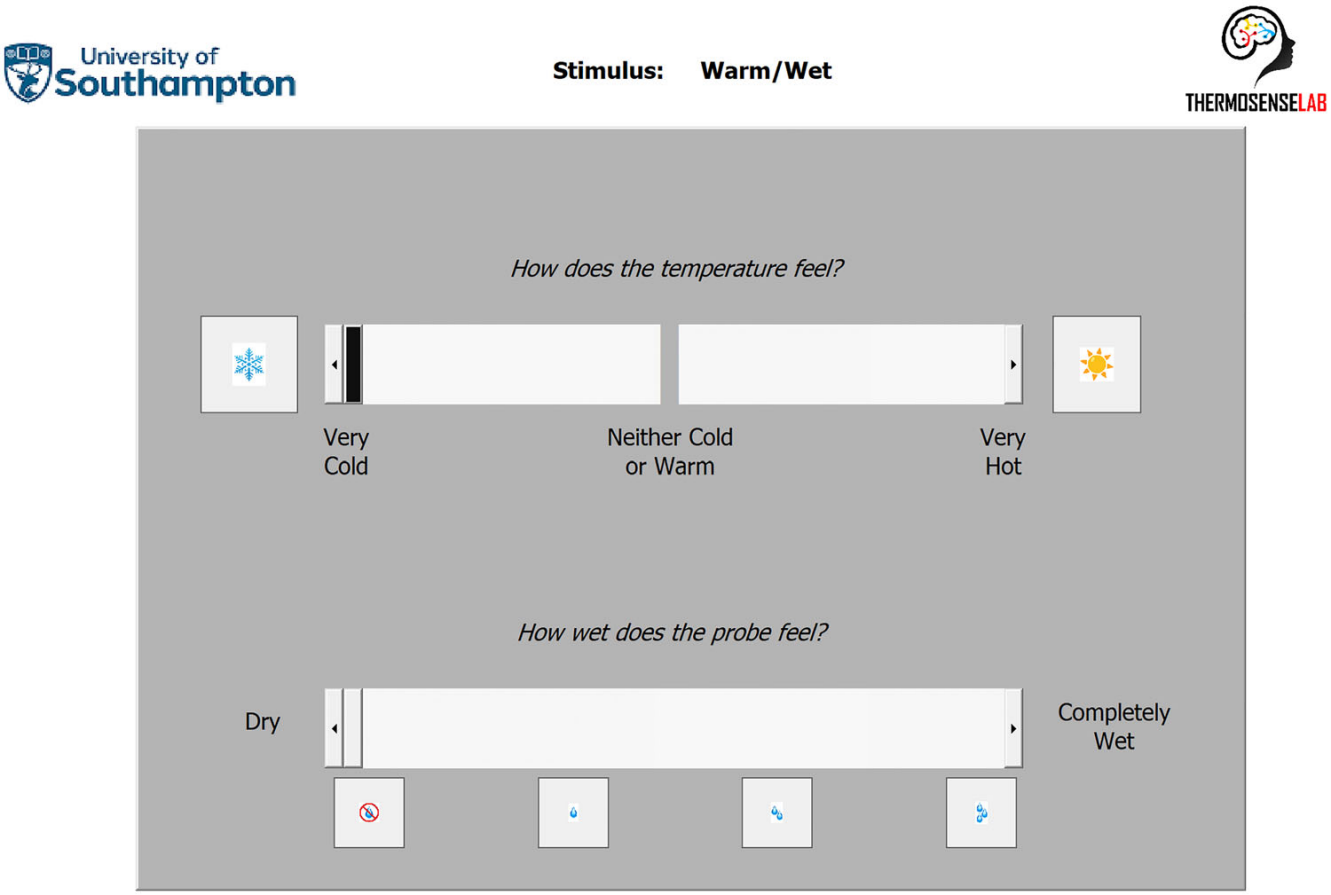
Visual analogue scale for wetness perception and thermal sensation used by all participants.

During each of the six stimulus applications, all participants were instructed to report their local wetness perception on our previously established digital visual analogue scale (length: 100 mm; anchor points: 0, dry; 100, completely wet) (Filingeri et al., 2014a; Valenza et al., 2019), which was modified to incorporate visual cues associated with wetness levels (following pilot studies with a subgroup of children) (Figure 1).

#### 2.3.2 Body mapping test

Following the multisensory integration test, participants underwent a body mapping test. This experiment involved participants reporting the perceived magnitude of local thermal and wetness perceptions arising from a single, short‐duration (i.e. 10 s) static application of a cold‐wet (i.e. 5°C below local skin temperature), neutral‐wet (i.e. equal temperature as local skin temperature) and warm‐wet (i.e. 5°C above local skin temperature) hand‐held temperature‐controllable probe (surface area: 1.32 cm^2^, water content: 0.8 mL; with 30‐s interval between stimuli). Participants reported the magnitude of their local sensations and perceptions on two digital visual analogue scales for thermal sensation (length 200 mm; anchor points: 0, very cold; 100, neither cold or warm; 200, very hot; Figure 1), and wetness perception (length: 100 mm; anchor points: 0, dry; 100, completely wet; Figure 1), respectively. We mapped thermal and wetness sensitivity at three different locations over the body: the centre of the forehead (i.e. 5 cm above the pupillary line), the posterior neck (i.e. over the process spinous of cervical 4), and the dorsal foot (i.e. midpoint between the second and third metatarsal joints). We chose those body regions because: (1) they present high exercise‐induced local sweat rates (e.g. forehead) (Smith & Havenith, 2012); (2) they are reported amongst the most thermally sensitive areas (e.g. neck) (Nakamura et al., 2013); and (3) they were recently reported to be more evidently impacted by ageing (e.g. foot) (Wildgoose et al., 2021).

### 2.4 Experimental protocol

Participants arrived at the laboratory on testing days and underwent preliminary measurements. They changed into shorts and T‐shirt before we marked the skin sites to be stimulated with a washable marker. Then we gently shaved the sites, where and when needed, to limit any insulative effect of hairiness on heat transfer during the application of the stimuli.

Following on this preparation, participants underwent 20 min of resting on a chair to adjust to the environmental conditions. During this time, all participants underwent familiarization with all testing procedures, including the use of the visual analogue scales (Valenza et al., 2019, 2024). Upon completion of this stabilization phase, the multisensory integration test commenced.

We first recorded the local skin temperature of the forearm with an infrared thermometer (Spot IR Thermometer TG54: FLIR Systems, Wilsonville, OR, USA). This parameter was used to determine the temperature of the first of six stimuli to be applied with a thermal probe (surface area: 1.32 cm^2^; NTE‐2A; Physitemp Instruments LLC, Clifton, NJ, USA), which was covered with a 100% cotton fabric. Depending on the moisture level required for each stimulus (see ‘Multisensory integration test’ section), the cotton fabric was either maintained dry (i.e. Neutral dry stimulus) or wetted with 0.8 mL of water using a pipettor. Following a verbal warning, the stimulus was applied statically on the participants’ testing site skin for 10 s, during which participants were instructed to report the magnitude of their very first perceived wetness using the visual analogue scale protocols (note that extensive studies in our laboratory have demonstrated peak wetness perception to be experienced within the first 10 s of contact) (Filingeri et al., 2014a; Valenza et al., 2019). Application pressure was not measured but was controlled to be sufficient to ensure full contact, at the same time not resulting in pronounced skin indention. Upon acquisition of the perceptual rating, we removed the stimulus, gently dried the skin with a paper towel using a single unidirectional stroke for all stimuli (including the dry one), and then repeated the same procedure for the remaining five stimuli based on a randomized order.

Once the multisensory integration test was completed, we allowed for a 10‐min break, before commencing the body mapping test. First, we recorded the local skin temperature of the first skin site to be tested (i.e. the forehead, neck or dorsal foot). We then prepared the first wet stimulus (e.g. cold‐wet, 5°C below local skin temperature) following the same procedures above (i.e. application of a water‐saturated cotton fabric). Following a verbal warning, the wet stimulus was applied statically on the participant's skin for 10 s, during which the participant was encouraged to rate both their thermal and wetness perception. Wetness perceptions were rated on the same visual analogue scale as per Figure 1. Thermal sensations were rated using an additional 200‐mm visual analogue scale (scale descriptors: 0 mm, very cold; 100 mm, neutral; 200 mm, very hot, Figure 1) previously used in our studies (Valenza et al., 2019, 2024). Upon acquisition of the perceptual ratings, we removed the stimulus, gently dried the skin, and then repeated the same procedure for the other stimuli (e.g. neutral‐ and warm‐wet) on the same skin site, before proceeding to the next skin site. The order of testing sites was counter‐balanced between participants; the order of testing stimuli (e.g. warm vs. neutral vs. cold wet) was counter‐balanced between and within participants.

### 2.5 Statistical analysis

First, we evaluated the independent and interactive effects of age (three levels: children vs. young adults vs. older adults), and type of stimulus (six levels: very cold‐wet vs. cold‐wet vs. neutral‐wet vs. neutral‐dry vs. warm‐wet vs. hot‐wet) on wetness perceptions recorded during the multisensory integration test, by means of two‐way mixed model ANOVA.

Second, we evaluated the independent and interactive effects of age (three levels: children vs. young adults vs. older adults), and body region (three levels: forehead vs. neck vs. dorsal foot) on wetness perceptions resulting from the cold‐, neutral‐ and warm‐wet stimuli during the body mapping test by means of two‐way mixed model ANOVAs (separately for each temperature stimulus). We used the same analytical approach to evaluate the independent and interactive effects of age and body region on thermal sensations.

In the event of statistically significant main effects or interactions, *post hoc* analyses were conducted with Tukey's test.

Normality testing using the Shapiro–Wilk test was performed for all datasets. Data are reported as the means, SD and 95% confidence intervals (CI). Observed power was computed using α = 0.05. Statistical analysis was performed using Prism, version 8.0 (GraphPad Software Inc., San Diego, CA, USA).

## 3 RESULTS

### 3.1 Multisensory integration test

**FIGURE 2:**
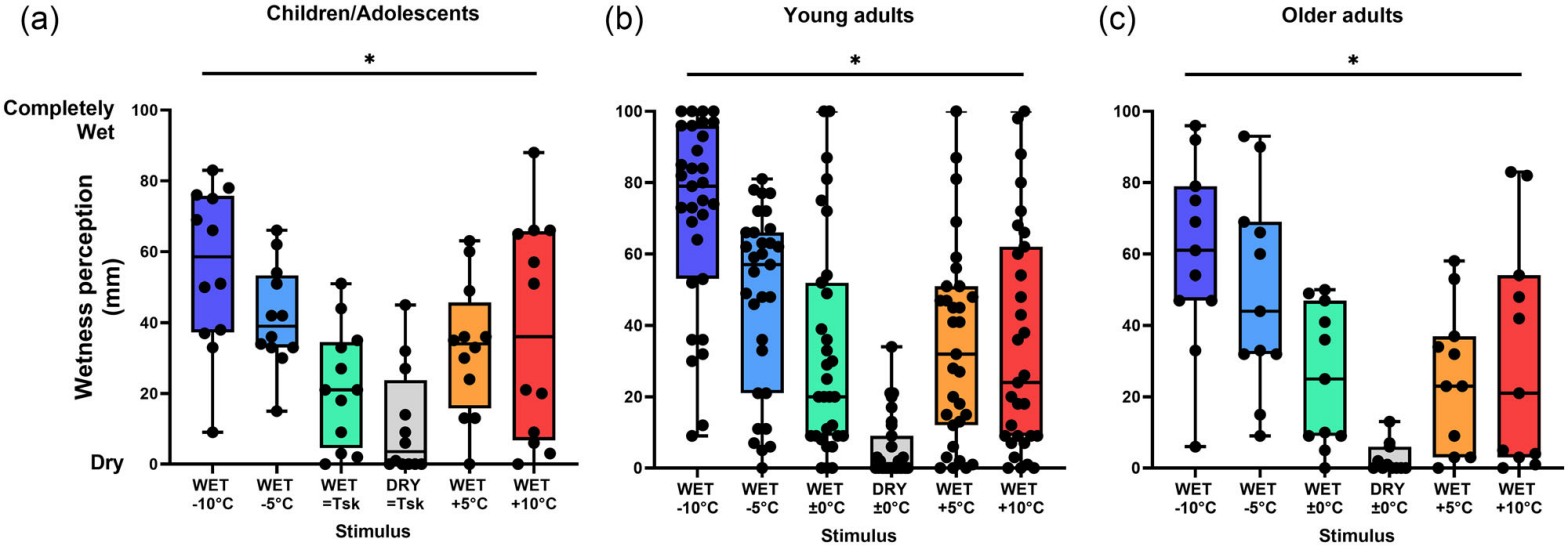
Box and whisker plots (min to max with interquartile range) presenting group and individual data for wetness perceptions recorded during six stimulus applications (i.e. either wet or dry at various temperatures relative to local forearm skin temperature, *T*_sk_) in 12 children (a), 41 younger adults (b) and 21 older adults (c). *Statistically significant main effect of stimulus temperature with *P *< 0.0001.

We found a statistically significant effect of type of stimulus (*F*_5,255_ = 35.44; *P *< 0.0001; 26% of total variance), but not of age (*F*_2,51_ = 0.68; *P* = 0.510), nor stimulus by age interaction (*F*_10,255_ = 0.68; *P* = 0.869), on wetness perceptions recorded during stimulation of the forearm. *Post hoc* analyses indicated that, despite all wet stimuli presenting the same moisture content, the children/adolescents (Figure 2a), younger (Figure 2b) and older adults (Figure 2c) perceived large temperature‐dependent differences in wetness perception. Specifically, we found that the very cold wet stimulus was perceived as wetter than the cold wet (mean difference: 16.1 mm [95% CI: 2.9, 21.4]; *P* = 0.006; ∼16% difference), the neutral wet (mean difference: 35.5 mm [95% CI: 22.3, 38.7]; *P *< 0.0001; ∼35% difference), the neutral dry (mean difference: 55.6 mm [95% CI: 42.7, 69.2]; *P *< 0.0001; ∼56% difference), the warm wet (mean difference: 31.7 mm [95% CI: 18.5, 44.9]; *P *< 0.0001; ∼32% difference) and the very hot wet stimulus (mean difference: 22.7 mm [95% CI: 14.5, 40.9]; *P *< 0.0001; ∼23% difference). We also found that the cold wet stimulus was perceived as wetter than the neutral wet (mean difference: 19.4 mm [95% CI: 6.1, 32.6]; *P *< 0.001; ∼20% difference), the neutral dry (mean difference: 39.8 mm [95% CI: 26.6, 53.1]; *P *< 0.0001; ∼40% difference), and the warm wet stimulus (mean difference: 15.6 mm [95% CI: 2.3, 28.8]; *P* = 0.009; ∼16% difference).

### 3.2 Body mapping test: wetness perception

**FIGURE 3:**
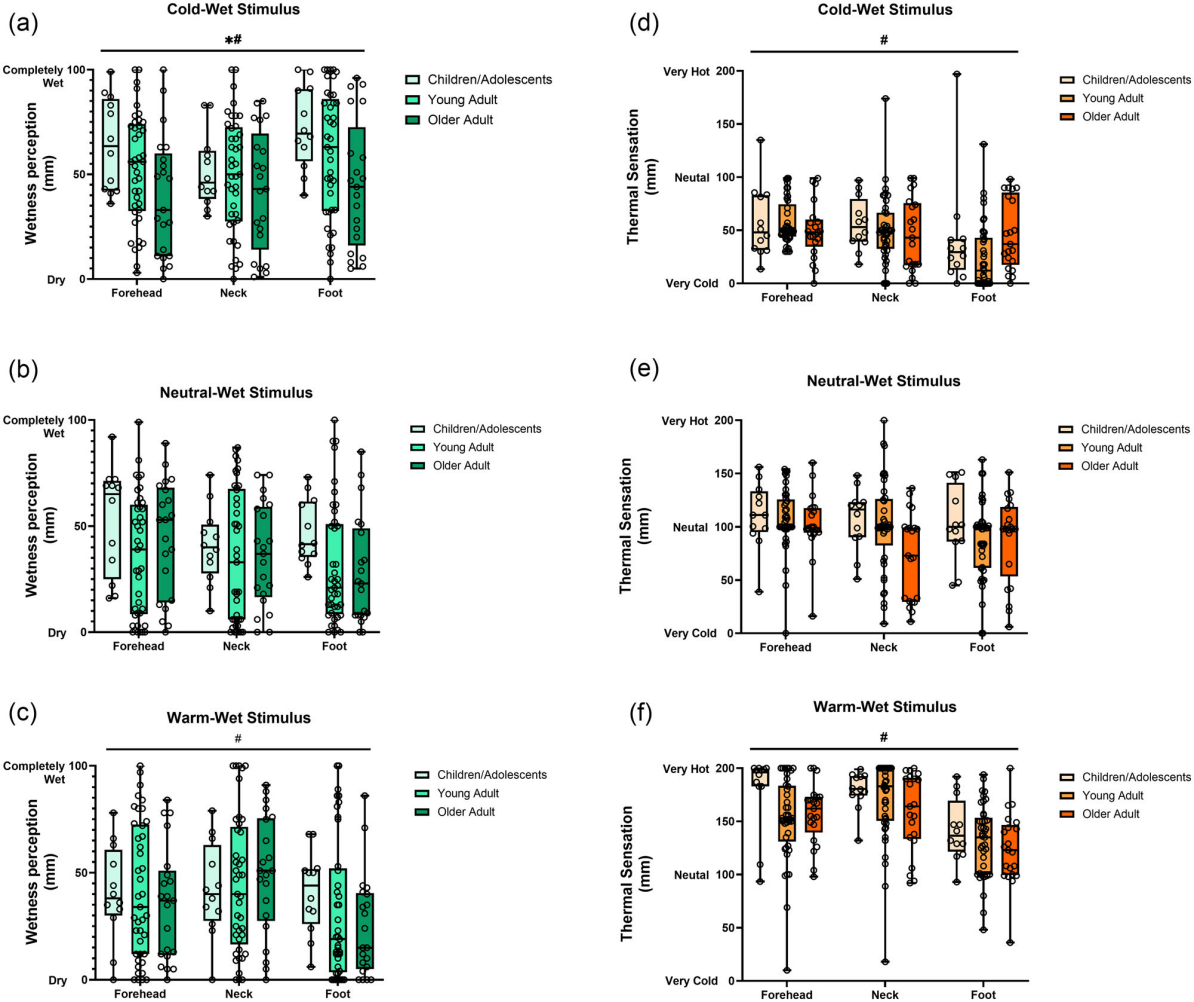
Box and whisker plots (min to max with interquartile range) presenting group and individual data for wetness perceptions (a–c) and thermal sensations (d–f) recorded during the application of cold‐wet, neutral‐wet and warm‐wet stimuli to the forehead, neck and foot dorsum in 12 children (a), 41 younger adults (b), and 21 older adults (c). *Statistically significant main effect of age (*P *< 0.05). #Statistically significant main effect of body site (*P *< 0.05).

When considering wetness perception arising from the application of the cold‐wet stimulus (Figure 3a), we found a statistically significant main effect of age (*F*_(2,71)_ = 3.71; *P* = 0.029), body site (*F*_2,142_ = 4.41; *P* = 0.014), but no interaction (*F*_4,142_ = 0.90; *P* = 0.464). *Post hoc* analyses indicated that, irrespective of skin site, children reported cold‐wet perceptions of greater magnitude than those of older adults (mean difference: 20.6 mm [95% CI: 1.5, 39.7] *P* = 0.031; ∼ 21% difference), and of similar magnitude to those of younger adults (mean difference: 8.7 mm [95% CI: −8.5, 26.0]; *P* = 0.451). Furthermore, *post hoc* analyses indicated that, irrespective of age, stimulation of the foot dorsum resulted in cold‐wet perceptions of greater magnitude than those resulting from stimulation of the neck (mean difference: 11.1 mm [95% CI: 2.2, 20.0]; *P* = 0.010; ∼11% difference).

When considering wetness perception arising from the application of the neutral‐wet stimulus (Figure 3b), we found no statistically significant main effect of either age (*F*_(2,71)_ = 1.97; *P* = 0.148) or body site (*F*_2,142_ = 2.35; *P* = 0.100).

When considering wetness perception arising from the application of the warm‐wet stimulus (Figure 3c), we found a statistically significant main effect of body site (*F*_2,142_ = 4.60; *P* = 0.012) but not age (*F*_(2,71)_ = 0.19; *P* = 0.826). *Post hoc* analyses indicated that, irrespective of age, stimulation of the neck resulted in warm‐wet perceptions of greater magnitude than those resulting from stimulation of the foot dorsum (mean difference: 12.9 mm [95% CI: 2.8, 23.0]; *P* = 0.008; ∼13% difference).

### 3.3 Body mapping test: thermal sensation

When considering thermal sensations arising from the application of the cold‐wet stimulus (Figure 3d), we found a statistically significant main effect of body site (*F*_2,142_ = 5.9; *P* = 0.003) but not age (*F*_(2,71)_ = 0.69; *P* = 0.501). *Post hoc* analyses indicated that, irrespective of age, stimulation of the foot dorsum resulted in colder sensations than those resulting from stimulation of the forehead (mean difference: 17.5 mm [95% CI: 4.9, 30.2]; *P* = 0.004; ∼17% difference) and of the neck (mean difference: 13.6 mm [95% CI: 1.0, 26.2]; *P* = 0.032; ∼14% difference).

When considering thermal sensations arising from the application of the neutral‐wet stimulus (Figure 3e), we found no statistically significant main effect of either age (*F*_(2,71)_ = 3.11; *P* = 0.051) or body site (*F*_2,142_ = 2.92; *P* = 0.057).

When considering thermal sensations arising from the application of the warm‐wet stimulus (Figure 3e), we found a statistically significant main effect of body site (*F*_2,142_ = 22.1; *P *< 0.0001) but not age (*F*_(2,71)_ = 2.97; *P* = 0.058). *Post hoc* analyses indicated that, irrespective of age, stimulation of the foot dorsum resulted in less warm sensations than those resulting from stimulation of the forehead (mean difference: 30.4 mm [95% CI: 16.3, 44.4]; *P *< 0.0001; ∼30% difference) and of the neck (mean difference: 37.0 mm [95% CI: 23.0, 51.1]; *P *< 0.0001; ∼40% difference).

## 4 DISCUSSION

The aim of this study was to investigate whether children and adolescents perceive skin wetness similarly to younger and older adults.

In relation to our first hypothesis, our results indicated that children and adolescents aged 7–16 years presented a U‐shaped relationship between stimulus temperature and perceived wetness magnitude like that observed in younger and older adults. As evidenced in Figure 2, all age groups perceived very cold‐wet stimuli applied to the forearm as wetter than cold‐wet, neutral‐wet, warm‐wet and very hot‐wet ones, despite all stimuli presenting the same moisture content (0.8 mL of water). Our group has previously provided empirical evidence in young adults for this characteristic U‐shaped relationship between stimulus temperature and wetness perception, and we have since described it as a hallmark of the neurophysiological mechanisms underlying skin wetness perception in humans (Filingeri et al., 2014a; Valenza et al., 2019). Specifically, our neurophysiological model of skin wetness perception predicts that, irrespective of the physical presence of moisture on the skin, cooling‐induced activation of cold‐sensitive A‐type skin thermoreceptors provides the strongest cue to trigger the neural representation of a typical wet stimulus (Filingeri et al., 2014a, 2015b). As a result, cold‐wet stimuli are more likely to be perceived as wetter than less cold ones. The previously observed asymmetrical nature of this characteristic U‐shape relationship (Valenza et al., 2019), whereby warmer wet stimuli may also induce wetness perceptions yet to a smaller extent than colder wet ones, was also evident in all age groups tested in this study (see Figure 2). Indeed, all age groups perceived very cold‐wet and cold‐wet stimuli as wetter than very hot wet ones.

Altogether, the observations above provide novel and compelling evidence that children and adolescents aged 7–16 years present adult‐like wetness perception responses; this may also indicate that children and adolescents (likely) rely on similar multisensory integratory mechanisms for the perception of skin wetness. Previous developmental studies focusing on the ability to centrally integrate tactile and visual cues have noted that optimal multisensory integration occurs relatively late in a child's development (Ernst, 2008), often above the age of ∼8 years (Gori et al., 2008; Nardini et al., 2008). Our current group‐level findings are aligned to these observations and extend them to a common multisensory experience such as the perception of skin wetness. We appreciate that a key limitation of our current study is that we did not have an equal distribution of children/adolescents above and below the age of 8 years. Hence, our study cannot directly address the question of exactly when the multisensory integration of skin wetness perception becomes adult‐like during development. Nevertheless, evaluation of individual responses in our younger children indicated that our youngest children (i.e. aged 7 and 9 years) also presented the characteristic U‐shaped, temperature–wetness relationship observed at a group level. Future studies could therefore consider utilising the multisensory integration test employed within this study, which appeared to be feasible in young children, to more directly interrogate this perceptual question in a larger cohort of children younger than 8 years old.

It is also important to note that our findings associated with our second experimental hypothesis provide further support to the likely presence of similar mechanisms of multisensory integration for wetness in children/adolescents. Indeed, and in relation to our second hypothesis, we found that children and adolescents presented regional differences in their perceived wetness across the body, which were like those observed in adults (see Figure 3). Specifically, we found that, within the constraints of the proximal (i.e. forehead and neck) and distal (i.e. foot dorsum) sites tested, all age groups reported greater wetness perception at the foot dorsum during cold‐wet stimulation, and at the forehead and neck during warm‐wet stimulation. These observations are aligned with our previously reported evidence on the heterogeneous, body‐regional distribution of skin wetness sensitivity (Valenza et al., 2019; Wildgoose et al., 2021); importantly they provide further evidence that such characteristic body region‐dependent differences are already well‐established in children and adolescents aged 7–16 years.

The third novel finding of this study, which relates to our second hypothesis, is that, irrespective of the skin site tested in the body mapping test, children and adolescents reported greater wetness during cold‐wet stimulation than older (∼ 20% difference), but not younger adults. Previous evidence on cold sensitivity in children, as acquired via detection threshold methods, has indicated that children below the age of ∼8 years present lower sensitivity to cold, which then increases to reach adult‐like levels around the age of 10 years (Blankenburg et al., 2010). Our findings appear somewhat in line with this previous evidence, as our 7‐ to 16‐year‐old children/adolescents presented similar levels of cold‐wet perceptions as the younger adult cohorts. As per the difference with the older cohort, visual inspection of data in Figure 2a provides qualitative evidence that sensitivity differences could have occurred steadily as a function of normal ageing (consider the forehead and foot sites in particular), which we know well is associated with somatosensory loss of function (Decorps et al., 2014; Guergova & Dufour, 2011). Furthermore, we noted that age‐dependent differences in cold‐wet perception were not as evident as a result of the −5°C cold‐wet stimulation during the multisensory integration tests (see Figure 2). We believe that this qualitative comparison across tests indicates a likely age by skin site interaction (e.g. colder sensitive regions such as those assessed during the body map tests may reveal great age differences than less cold sensitive regions such as the forearm). Once again, we appreciate that a key limitation of our current study is that we did not have an equal distribution of children/adolescents above and below the age of 8 years. Hence, our study cannot directly address the question of exactly when the body regional patterns of skin wetness perception become adult‐like during development. Future studies could therefore consider utilising the body mapping test employed within this study, which appeared to be feasible in young children, to more directly interrogate this perceptual question in a larger cohort of children younger than 8 years old.

### 4.1 Conclusions

We conclude that wetness perceptions in children/adolescents (age range: 7–16 years) are similar to those of adults in that both present (1) a characteristic U‐shaped relationship between stimulus temperature and perceived wetness magnitude and (2) similar body regional patterns. This novel evidence opens the question of whether the perception of skin wetness is a ‘hard‐wired’ perceptual process based on a typical representation of wet stimuli, which humans may acquire early in their development to accommodate the lack of a skin hygroreceptor. These findings also provide novel evidence on age‐dependent variations in wetness perception, which could inform user‐centred innovation in thermal protection and garment design that accommodate the comfort needs of different age groups.

## AUTHOR CONTRIBUTIONS

Alessandro Valenza, Antonino Bianco, Davide Filingeri, and Peter R. Worsley conceived and designed the research. Alessandro Valenza, Hannah Blount, Jade Ward, Charlotte Merrick, Riley Wootten, Jasmin Dearden, Charlotte Wildgoose, and Alex Buoite‐Stella collected the experimental data. Alessandro Valenza, Hannah Blount, and Davide Filingeri analysed the data and drafted the manuscript. All authors revised the manuscript for intellectual content. All authors have read and approved the final version of this manuscript and agree to be accountable for all aspects of the work in ensuring that questions related to the accuracy or integrity of any part of the work are appropriately investigated and resolved. All persons designated as authors qualify for authorship, and all those who qualify for authorship are listed. For the purpose of open access, the author has applied a Creative Commons attribution license (CC BY) to any Author Accepted Manuscript version arising from this submission.

## CONFLICT OF INTEREST

None declared.

## Data Availability

The data that support the findings of this study are available from the corresponding author upon reasonable request.
